# Perceptions of inspiratory muscle training in adults recovering from COVID-19

**DOI:** 10.1371/journal.pone.0270620

**Published:** 2022-11-03

**Authors:** James Shelley, Joanne Hudson, Kelly A. Mackintosh, Zoe L. Saynor, Jamie Duckers, Keir Lewis, Gwyneth A. Davies, Ronan M. G. Berg, Melitta A. McNarry

**Affiliations:** 1 Applied Sports, Technology, Exercise and Medicine Research Centre, Swansea University, Swansea, United Kingdom; 2 School of Sport, Health and Exercise Science, Faculty of Science and Health, University of Portsmouth, Portsmouth, United Kingdom; 3 All Wales Adult CF Centre, Cardiff and Vale University Health Board, Cardiff, United Kingdom; 4 Hywel Dda University Health Board, Carmarthen, United Kingdom; 5 School of Medicine, Swansea University, Swansea, United Kingdom; 6 Faculty of Health and Medical Sciences, Centre for Physical Activity Research, and Department of Clinical Physiology and Nuclear Medicine, University Hospital Copenhagen–Rigshospitalet, University of Copenhagen, Copenhagen, Denmark; Universita degli Studi di Milano, ITALY

## Abstract

Post COVID-19 condition can occur following infection with SARS-CoV-2 and is characterised by persistent symptoms, including fatigue, breathlessness and cognitive dysfunction, impacting everyday functioning. This study explored how people living with post COVID-19 experienced an eight-week inspiratory muscle training (IMT) rehabilitation programme. Individualised semi-structured interviews with 33 adults (29 female; 49 ± 10 years; 6–11 months post-infection) explored expectations of IMT prior to the intervention, and post intervention interviews explored perceptions of IMT and its impact on recovery. Inductive thematic analysis was used to analyse the data. IMT helped many to feel proactive in managing their symptoms and was associated with perceived improvements in respiratory symptoms, exercise and work capacity, and daily functioning. IMT was well perceived and offers significant potential for use as part of a holistic recovery programme, although it is important to consider the complex, varied symptoms of post COVID-19, necessitating an individually tailored rehabilitation approach.

## Introduction

The World Health Organization (WHO) definition for individuals experiencing long-term symptoms following infection with Severe Acute Respiratory Syndrome Coronavirus 2 (SARS-CoV-2), often referred to as ‘long-COVID’, identifies that this condition occurs usually 3 months from the onset of coronavirus disease-2019 (COVID-19) symptoms and follows a probable or confirmed diagnosis. Symptoms last for at least two months and include fatigue and shortness of breath, will not necessarily mirror those experienced during acute infection and whilst they might fluctuate and relapse, will likely impact on daily functioning [1]. This definition not only captures the heterogenous nature of the condition and the variety and fluctuations in symptoms, but also highlights its potential impact on activities of daily living (ADLs). As of 1^st^ February 2022 there have been more than 370 million confirmed cases of COVID-19 globally [2]. In the United Kingdom (UK), ~10% of patients who have tested positive for SARS-CoV-2 virus report persistent symptoms beyond three weeks, although this is likely an underestimate as testing has not been widely available as a prerequisite for a COVID-19 diagnosis throughout all of the pandemic [3]. It is estimated that 1.1 million people in the UK have experienced persistent symptoms for more than four weeks following SARS-CoV-2 infection, with 831,000 reporting symptoms beyond 12 weeks [3]. Along with fatigue and cognitive dysfunction, breathing issues are reported as the most debilitating symptom associated with post COVID-19 [4].

With the potential for mutations to lead to new strains, as already observed with the Delta and Omicron variants, COVID-19 continues to represent a significant healthcare challenge, thus heightening the need for more understanding of how to support long-term recovery. There is currently a lack of established treatments and rehabilitation strategies for individuals recovering from COVID-19. However, given the heterogenous nature and novelty of the condition, rehabilitation requires careful prescription and monitoring, and in some cases may include an emphasis on physical activity as part of a person’s day-to-day life, rather than conventional structured exercise training [5]. Whilst graded-exercise therapy is typically a core component of pulmonary rehabilitation, based on the evidence currently available it is not recommended for individuals recovering from COVID-19 due to the presence of post-exertion symptom exacerbation [5]. This therefore highlights the requirement for alternative rehabilitation strategies. A survey of Physiotherapists in Australia reported that respondents had a perceived lack of resources and experience to use rehabilitation devices; despite this, among the experienced respiratory physiotherapists surveyed 77% were using respiratory muscle training devices as part of their therapy [6].

Inspiratory muscle training (IMT) uses a resistive breathing device to elicit improvements in respiratory muscle strength and endurance [6]. The inspiratory muscles, including the diaphragm, are morphologically and functionally skeletal muscles and respond to training in the same way as peripheral musculature [7]. IMT is associated with improvements in dyspnoea and quality of life in individuals with chronic obstructive pulmonary disease [COPD; 8] where diaphragmatic and skeletal dysfunction is common. Although the mechanisms of breathlessness are not yet fully understood in COVID-19, dyspnoea and impaired QoL are both commonly reported [4] and may be improved using IMT as part of a COVID-19 rehabilitation programme.

The National Institute for Health Research recently highlighted a need for research to enhance understanding of the recovery from COVID-19 by listening to the lived experiences of people living with post COVID-19 [5]. Shelley et al. [9] recently reported the impact on activities of daily living, including physical activity and work, highlighting the difficulty people with post COVID-19 experienced engaging in physical activity and exercise, and frequent cycles of increasing physical activity followed by exacerbations of symptoms. A profound impact was also cited on activities of daily living and people’s ability to work. Given these impacts and the challenges associated with managing the range of symptoms associated with the condition, it is perhaps understandable that symptoms of anxiety and depression were also reported, with others describing frustration from dealing with uncertainty and the length and variability of their recovery. Given the multifaceted nature of post COVID-19, it is especially important to involve participants in the exploration of attitudes and perceptions in order to enable the development of complex interventions [10]. A recent scoping review of rehabilitation programmes for individuals recovering from COVID-19 also highlighted the requirement for further qualitative research to compliment the growing body of clinical research [11]. Despite this there is a lack of research exploring perceptions of people undergoing interventions seeking to enhance recovery from COVID-19. This study therefore aimed to explore individuals’ experiences of using IMT to support their recovery from COVID-19.

## Methods

### Study design

Our methodological orientation embraced a naturalistic perspective, examining the individuals’ experiences of the phenomenon in question (here, experiences of IMT as a technique to support recovery from COVID-19) in its natural state [12]. We used qualitative description [13, 14], seeking to provide a rich, direct description of individuals’ experiences of the training and did not adopt an apriori theoretical perspective, as is common in qualitative description [15]. The consolidated criteria for reporting qualitative research was used to ensure explicit and comprehensive reporting (S1 File).

### Participants

Forty-eight adults recovering from COVID-19 (41 female; 47 ± 10 years; BMI 27 ± 7 kg·m-^2^) who were 6–11 months post-COVID-19 infection were recruited from a larger sample (*n* = 281), participating in a randomised control trial investigating the effects of an eight-week IMT programme (Clinical trial number: 48075; Health and Care Research Wales Portfolio), 33 (29 female; 49 ± 10 years; BMI 27 ± 8 kg·m^-2^; S2 File) of whom attended both pre- and post-IMT interviews.

Briefly, sampling for the initial RCT was purposive and participants were recruited via social media, online COVID-19 support groups, or following hospital discharge, and were eligible to participate if they were ≥ 18 years and had a previous infection with SARS-CoV-2, albeit a positive polymerase chain reaction test from an oral or pharyngeal swab was not mandatory, due to inconsistent testing availability in the UK at the time of their infection during the early phases of the pandemic. Standard pulmonary rehabilitation exclusion criteria were applied, excluding individuals with: i) dementia to the extent that they could not follow commands/training; ii) unstable cardiac disease, myocardial infarction or non-ST-elevation myocardial infarction within six weeks.

### IMT programme

The IMT programme required participants to complete three sessions of training per week for eight weeks; each session consisted of up to six sets of six breaths. The experiences of COVID-19 and, specifically, its impacts on physical activity, work and daily living, prior to engaging in IMT, of 14 of these participants have previously been reported, 9 of whom are also included in this study [9].

### Data collection

Ethics approval was granted by the London Centre Research Ethics Committee (Ref: 20/HRA/3536) and College of Engineering, Swansea University Research Ethics Committee (2020–037). Participants were fully informed of the aims of the research and provided written and verbal consent prior to participation. Individual semi-structured interviews were conducted prior to and following the intervention (pre-intervention interviews lasted between 20 and 64 minutes (mean 38 ± 11 minutes) and post-intervention interviews lasted between 17 and 48 minutes (mean 30 ± 7 minutes).

### Interview guide

The initial interview guide was developed by JH and aimed to uncover participants’ perceptions of the IMT, its perceived impact on them and their lifestyle, practical considerations about using the device, and, recommendations for using the device in the future. Opening questions were outlined for each topic, with follow up questions to obtain more detail where appropriate, and, prompts to facilitate responses, as needed. JS reviewed and modified the guide to ensure that the interview questions were comprehensive and comprehensible. All interviews were conducted and recorded via online video conferencing software (Zoom Video Communications, San Jose, CA) by a trained researcher (JS, PhD; a male postdoctoral researcher employed exclusively on this project at the time with training in conducting qualitative interviews and research interests in clinical exercise physiology), with participants and researcher in their own homes, and transcribed verbatim. No-one else was present during interviews.

### Data analysis

We followed principles of reflexive thematic analysis in our data analysis, involving deep and prolonged immersion in the data, using a fluid and recursive, but nevertheless rigorous and systematic, process to actively generate themes [16].

The primary analyst (JS) conducted all the pre and post-IMT interviews and had detailed knowledge of the potential effects of IMT. He also managed the delivery of the IMT to participants; during which JS recorded his ongoing thoughts and reflections about the sample and their engagement with the IMT, based on informal observations he made during these encounters with participants. He was therefore deeply immersed in the data over a prolonged period of time and had an opportunity to develop a strong rapport and relationship with the participants. This reflective process enabled him to be aware of, and share, his biases regarding IMT as a result of his role and prior knowledge. During the analysis process, he drew on these reflections to inform discussions about the data with the other analysts, supporting the rigor of this process.

Using the steps identified by Braun and Clarke [17, 18] as guidelines, first, to become familiar with the data, JS and JH read and re-read pre and post-IMT interview transcripts to gain a sense of each participant’s personal experience of COVID-19, their motives and expectations for completing the IMT, and, how they experienced IMT. JS used NVivo 12 (QSR International) to organise and inductively code the data, adopting a constructivist approach to examine realities, meanings, and experiences of individuals recovering from COVID-19 and participating in an eight-week IMT intervention. Following this, using these codes, he then developed themes to represent patterns in the data that describe people’s experiences by first collating codes with shared content into sub-themes and then combining similar sub-themes into overall themes.

JH reviewed the sub-themes and themes, acting as a critical friend to query the coding of the data and the themes that were generated. She completed this process independently and then through dialogue with JS, enabling themes to be refined, named and organised into a thematic map. This dialogue also explored how her biases, resulting from prior experience as an exercise psychologist and qualitative researcher, were impacting on her interpretations of the themes. MM, an exercise physiologist with clinical trials experience and who had recruited and informally discussed their experiences with many of the participants, reviewed the thematic map and quotations used to illustrate the generated themes leading to suggestions about which did not seem to fit together and which required further clarity.

**Fig 1:**
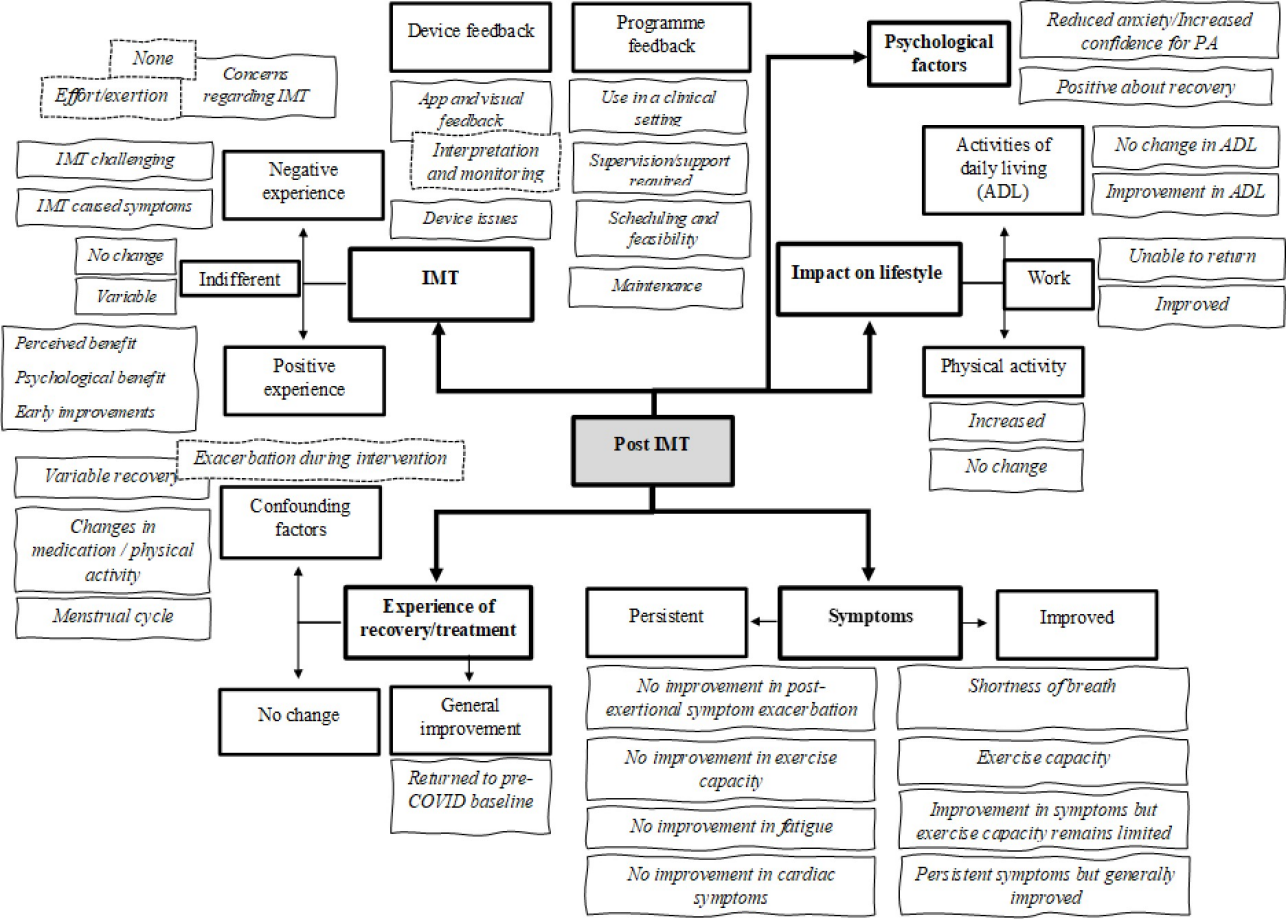
The five overarching themes (bold text and box), sub-themes (standard font and bold box), codes (italic text) and related codes (italic text and dashed box) identified from semi-structured interviews exploring individuals’ experiences of COVID-19 recovery following an 8-week inspiratory muscle training intervention (IMT = Inspiratory Muscle training; COVID-19 = Coronavirus disease-2019; PA = Physical Activity; ADL = Activities of Daily Living).

Following this discussion, JS and JH reviewed and refined the themes, continuing this process throughout the writing of the manuscript until its final iteration, as presented in the thematic map shown in Fig 1. Multiple voices were therefore represented in the interpretation of the data, from participants and analysts.

### Quality

Our analysis processes, as described above, incorporated critical elements of quality reflexive thematic analysis [18, 19]. We immersed ourselves in the data through prolonged engagement with the transcripts and repeated and prolonged engagement with participants. We examined our biases and assumptions throughout the full analysis process, for instance, independently, in reflexive note-taking (JS) and collectively through discussion, enabling us to challenge interpretations and explore and reflect on the influence of our experiences and different roles. We reviewed our codes and themes independently and through critical debate, returning to the data and the meaning behind participants’ comments, checking that quotes, codes and themes were coherent, and themes reflected people’s experiences.

## Results

Five overarching themes were constructed post-intervention (Fig 1); where relevant, these themes are discussed in relation to pre-intervention to provide the context for the intervention and to comment on any changes participants reported.

### Pre-IMT intervention

#### An unpredictable, unexpected and unique experience

As the pandemic progressed, understanding of the variety of symptoms associated with COVID-19 improved. However, participants experiencing these symptoms in the early stages of the pandemic were presented with significant challenges and concerns. Whilst it is beyond the scope of this article to discuss the full extent of cardiovascular, respiratory and neurological symptoms experienced by participants, it is pertinent to note the variability in symptoms participants experienced in the months prior to the intervention and the challenges associated with this:

“*Every time I think I’m getting better it’s like Bam*! *another relapse*… *Literally they’re coming about every 4–7 days*, *the relapses*. *Some last a few hours*, *sometimes they’ll last a few days*, *sometimes they last a few weeks*. *And there are varying degrees of severity*. *Sometimes I’ll have to go back to bed during the day and other times I’ll just take a little power nap and then I can plough through”* (P061)

Often ‘relapses’ or exacerbations of symptoms were associated with exertion:

*“I’m scared of relapsing again because*, *if I push myself too hard*, *I relapse*, *that’s what’s happened in a kind of steady cycle*, *over the last four months or so”* (P015)

Whilst there was a range of symptoms reported, the most common were shortness of breath, reduced exercise capacity and fatigue:

“*what’s persisted for me really is the tight chestiness*, *the cough and the fatigue*. *Most of the other things have mostly cleared*. *I’m now at*, *it’s eight months tomorrow”* (P042)

The impact of these symptoms on individuals’ physical activity, exercise, activities of daily living and work were broadly similar to those identified in our previous analysis Authors (2021), with participants reporting being limited by their symptoms and finding engaging in any form of physical activity difficult:

“*I became really scared of doing any exercise because of the way it would impact me for days afterwards”* (P044)

#### Taking responsibility for recovery

Participants discussed why they volunteered to participate in the study, citing that the IMT provided them with an opportunity to be proactive about their rehabilitation:

*“I’m not going to get any help from the NHS [National Health Service] … so it’s about self-help and if you’re doing this trial*, *that might actually help me and I was interested in it”* (P008)

“*…if there’s anything I can do that’s going to help then it’s worth doing isn’t it … I’m doing as much as I can in terms of resting and doing gentle activity*, *like the doctors are saying but actually I’d rather try to be proactive and do something that might help”* (P027)

Many participants hoped that the IMT would help them to increase their physical activity levels and potentially return to their usual activities:

“*I think this is probably a good way for me and try and build fitness as well in a more kind of sustained*, *controlled way and I’m keen to do that”* (P005)

Other motives for participating included receiving some additional support with their recovery as well as helping to contribute to the understanding of COVID-19 by participating in research:

“*I think*, *again*, *because being physically healthy and physically fit has been such an important part of what I’ve done in the past and the fear of*, *how do I get back into shape and what’s safe and what isn’t*?*… I feel like it would help to have more guidance on how to get back into a physically fit state*” (P068)

“*I just think at the moment there’s so much that’s unknown about COVID and I think*, *you know*, *I’ve had COVID so I kind of feel that I want to participate in as much research as I can to help the people that are doing the research kind of understand a bit more about the condition so it can help the next group of people that have got it*. *So*, *for me that feels quite important”* (P037)

Some participants did express some concerns with taking part in the research and completing the IMT programme, but were willing to attempt it:

“*I am concerned there may be payback for doing it but I’m willing to take the risk because I can’t do nothing”* (P058)

### Post-intervention

Following the intervention, participants shared their experiences of using the IMT device and any perceived impact on their recovery, represented by five themes: on the right road but still travelling; breathlessness was only part of the puzzle; impact on lifestyle; regaining confidence and positivity and how did people experience IMT?.

#### What did IMT offer the individual?

*On the right road but still travelling*. Following the eight-week IMT intervention, the participants reported feeling that their general health and COVID-19 symptoms had improved:

“*Doing a lot better*. *Sort of energy-wise a lot the sort of fatigue has really improved and breathing-wise I have noticed a big improvement as well*. *So*, *I am still having some episodes of getting sort of short of breath but it is not as often and I am able to do a lot more kind of before I get to that point”* (P037)

For others, the improvements were slight but still noticeable:

“*COVID symptoms probably not totally dissimilar to when I saw you*, *definitely a bit of an improvement though in everything*, *so I’m able to do slightly more overall”* (P025),

whereas some participants reported no change in their condition over the eight-week period:

“*I feel like I’ve probably not really improved since I last spoke to you*, *however*, *I’m not worse”* (P033)

Nevertheless, participants also acknowledged the difficulty in assessing their recovery owing partly to the variable nature of their condition and to the various other treatments they were using including pacing, exercise and vaccinations which may also be contributing to their improvements:

“*It’s hard because everything is really slow*. *You feel a setback happens*, *but you maybe don’t go back as far as you were before but it’s all back and forth*. *So*, *it’s quite hard to judge it for yourself”* (P024)

In addition, some participants also experienced exacerbations of their symptoms during the intervention which hindered their ability to complete the training and made it challenging to assess their recovery during this period:

“*I started the inspiratory muscle training and I felt like it was working my muscles when I started which was good*. *In between then*, *I had some relapses and I was diagnosed with myocarditis which they thought I’d previously had before*. *Then I got a bit worse*, *so the cardiologist decided to treat me for that with some tablets which made me feel better actually”* (P016).

#### Breathlessness was only part of the puzzle

The primary aim of the IMT was to improve respiratory symptoms, and following the intervention participants consistently reported experiencing less shortness of breath:

“*I genuinely think it has helped my breathing*. *I think it has made me*, *take a bigger breath*, *I don’t feel as breathless*, *I feel stronger…”* (P007)

Participants also perceived a positive impact of IMT on their physical fitness and capacity to engage in physical activity and exercise:

“*I just feel fitter really… I’m just able to do things which I*, *I wasn’t doing before and I’m not a hundred percent there but I’m sort of ninety percent back to where I was*, *so that’s really good”* (P040)

Whilst improvements in respiratory symptoms were reported, for many these were not the primary symptoms impacting their day-to-day life, with some participants still experiencing fatigue and post-exertional symptom exacerbation:

*“I don’t really notice feeling breathless anymore*. *I think that is the thing that has changed but in terms of it affecting my fatigue which is sort of the thing that is actually affecting my life the most*, *that hasn’t really changed at all”* (P044)

“*… as my breathing’s improved*, *I’ve then pushed myself harder to go further but then what I’m finding is I hit this glass ceiling where my other issues like this kind of joint pain and relapse and fatigue kicks in because I’m overdoing it*” (P027)

#### Impact on lifestyle

Participants discussed the impact that COVID-19 was still having on their lifestyle following the eight weeks of IMT, including the impact on physical activity, ADL and work.

#### Building back to baseline

Some participants were starting to return to their previous lifestyles, reporting an increase in physical activity and exercise post-intervention:

“*… there’s a specific walk I wasn’t able to do two or three months ago*, *that I can do now without having a bad effect… or regretting my decision*, *wishing I hadn’t gone that far*. *It’s still not as far as I would go under normal health*, *but it’s certainly an improvement on what I was doing before”* (P024)

Similarly, many participants reported that they were able to do more ADL and with greater relative ease:

*“I’m managing to keep on top of things around the house and doing*, *you know*, *tiny chunks of gardening … I’m finding I’m resting less in the day… I can do more of just pottering around in the day*, *whereas before*, *I was having to rest and sit and do nothing for big chunks*. *So*, *it’s a lot more of my day spent doing things”* (P064)

As with prior to the intervention, participants’ employment situations varied, with some participants being able to return to work during or following the intervention:

*“… in terms of my work as well I am able to do a lot more which is fantastic*, *which is what I wanted to do to be able to do more work and I feel I have got the energy…”* (P017)

#### Stuck in the cycle

Not all participants experienced these improvements following the intervention, with some reporting little change in their physical activity levels and exercise:

*“I was getting breathlessness and that’s less now but it wasn’t the limiting factor for how much I could do, so it was a bit like when that’s not the limiting factor, even though it might help the breathlessness, how much is that actually going to impact on activity*

*level*?*”* (P025)

Whilst others had returned to work but found it challenging and a number of participants reported being unable to return to work:

*“I was feeling quite good*, *well reasonably good*. *Basically*, *it’s gone downhill since I’ve gone back to work”* (P033)

“*In terms of work*, *since December*, *I am off sick… and I am not ready to go back in that way*. *So*, *I might be losing the job”* (P050)

#### Regaining confidence and positivity

Following the intervention some participants described improvements in their mood:

“*I would say my mood is way*, *way better than before I started*. *It has had a definite impact*. *Partly for having something to do*, *partly for somebody recognising that this is something that is happening and something to focus on so*, *so yeah a big difference”* (P001)

Participants also reported reduced anxiety about feeling short of breath and the limitations this could have on their daily activities. With this some participants also reported increased confidence to engage in physical activity following the intervention:

“*I would say I feel*, *I don’t feel anxious before exercise which I know I mentioned to you I did previously*, *so that has gone*. *So that is huge actually to not feel anxious in my daily life”* (P001)

“*I felt really nervous of getting short of breath*, *I remember*, *it was probably around the time you and I first talked*, *I remember looking up a small hill*, *near where I live*, *thinking*, *oh God*, *I’ve got to walk up there and I did and I was alright but I felt really nervous and I think that*, *I feel more confident in what I can do”* (P042)

#### How did people experience IMT?

*Inspiratory muscle training*. Participants provided feedback related to their overall experience of the IMT programme, and gave specific feedback on using the device and app. They also commented on the potential use of IMT in clinical practice to support recovery from COVID-19.

#### Challenging but worth the effort

For many, participating in the IMT intervention was a positive experience:

“*I think that it is*, *it has just been fantastic*. *Yeah*, *I feel*, *I feel so different that in a very positive way mentally and physically and emotionally*, *you know and cognitively*, *just the whole thing”* (P017)

“*I think that that definitely made a positive difference because if I compare sort of the last three months compared to the three months before*, *to me there has been a marked change*. *So*, *I think it has definitely made a difference yeah”* (P037)

Despite this, some participants reported discomfort which improved throughout the programme and they did not perceive it to be a barrier to completing the training:

“*I think initially they [symptoms] got worse and I think that was just simply because I was doing something new and it was a bit exhausting at the beginning and I think that made the relapses a bit worse*, *but I’m stubborn*, *I wanted to you know*, *improve when I felt I needed to improve and I wanted to carry on with the training*, *it didn’t put me off”* (P061)

#### Tedious and Tire(d)some

Some participants remained somewhat indifferent to having completed the IMT and were neither in favour nor against the training:

“*I felt*, *to begin with I definitely felt it was helping but ultimately I’m not sure whether it has helped or not really”* (P038)

There was also a number of participants who reported having a negative experience of the IMT, primarily due to finding the training challenging. For some the IMT became tedious and it was perceived that the required time and energy could be used on other activities

“*I did start to feel a bit more negative about it*, *it just started to feel like a bit of a chore*, *because of my fatigue*, *it feels like time is really precious because I don’t have as much time in the day as I used to”* (P044)

Others did not perceive the sensation of exerting themselves and the accompanying discomfort positively:

“*I’d get these pains and things after I’d done it and I was tired after I’d done it as well and it’s hard because you know*, *you’re sitting on a chair sort of obviously doing the breathing thing but I didn’t expect to feel as tired as I did…”* (P046)

#### Ins and outs of using the PrO2

Participants commented on the ease of use of the PrO2 IMT device and particularly the compact and portable design which meant that the device could be used remotely:

“*I quite liked it*, *it was quite a neat*, *it was easy to hold and easy to use*. *The device was fine”* (P078)

“*I was able to do this in the comfort of my own room*, *I didn’t have to go anywhere*, *I didn’t have to*, *you know*, *make appointments every single week”* (P068)

Some participants experienced problems with their devices, including issues charging the device and some devices breaking during the intervention; these issues were subsequently resolved but caused disruption:

“*I’d put it on charge and then randomly it just wouldn’t hold charge and I’d have to sort of keep it plugged in*, *while I was doing the actual training*, *which made it a bit sort of tricky”* (P040)

“*The initial PrO2 … it worked like once or something and then I wasn’t really*, *I wasn’t able to turn it on*, *so you send me a second one… so I think I lost a week or two there*, *just trying to figure it out and then I got the new one and started using that one”* (P068)

The PrO2 device worked via an app downloaded on a compatible device which provided biofeedback on every inspiration. Participants commented on the ease of use of the app as well as the information provided via the app. For many the app was easy to use:

“*I mean*, *the app was fine*, *taking through very straightforward*. *Very easy to use*, *really”* (P050)

However, a number of participants experienced connectivity problems and issues with the user interface:

“*The app was*, *you know*, *quite poor I felt*, *it often took a lot of time for my device and my mobile phone to connect… I mean the graphics are dreadful… I found it quite glitchy sometimes on the App and it quite often crashed out…”* (P038)

The purpose of the app was to provide biofeedback to participants to guide them through the IMT programme. This feedback was well received by the majority of participants:

“*It was good to see*, *I think you need something that when you are doing it you get a little graph and then the numbers go green when you hit your*, *what you should be going for”* (P007)

“*I think the gamification of it with the power curves etc*. *was great*. *I think being able to see week-on-week improvements is obviously an inspiration as well*, *you push yourself”* (P008)

The issues with the feedback primarily related to finding it difficult to understand or interpret the information provided, particularly when attempting to track progress over time:

*“I looked at the app every time I used it but I didn’t understand the figures and I suppose I didn’t really take the time out to understand them but it wasn’t obvious what was happening…”* (P010)

“*…the graphs are of each session*, *I think*, *and it would be nice to be able to see in a graph form*, *in a visual form*, *have these upward or downward trend or whatever is happening with each session”* (P044)

#### Real world application

The vast majority of participants stated that they would recommend IMT for individuals recovering from COVID-19:

"*… like I say*, *anybody who’s has COVID should speak to a GP and mention this as a possible benefit*, *they should go for it*, *definitely"* (P008)

Participants also provided recommendations for how IMT could be used in a clinical setting. The intervention was unsupervised and although participants were able to contact a researcher at any point, the feedback provided following the intervention suggested that participants would have liked additional support during the intervention:

“*I didn’t have any contact in that eight-week period so maybe a little touch base halfway might be good*. *Just motivation and making sure there was no problems”* (P007)

“*…maybe it would have been good to say*, *maybe the results of what you’re doing… so you get some feedback on that*. *I know this is a study so it’s different*, *but if I was doing it through physio*, *I’d expect to be seeing them”* (P024)

Participants suggested that it would be appropriate to access the IMT device through a GP referral or for those who had access, via a COVID clinic:

“*…either the GP or if they get referred to Long-COVID clinic*, *you know*, *having them [PrO2]… there as well as something that they could offer and train and follow-up on would probably be quite useful too”* (P038)

Participants also acknowledged that individuals may benefit from IMT at different times during their recovery and that consideration of their symptoms, primarily fatigue and shortness of breath, should form part of the decision making before starting IMT:

“*… I think if they did it*, *if there are these proper COVID clinics because hopefully they would be looking at you holistically so they’d be able to take all the tests*, *look at where your body’s at and fit it into a whole package of whatever they’re doing you know and it might be right for some people and it might not be right for others and putting it as part of a holistic package would make more sense to me”* (P036).

Participants were 6 to 11 months into their recovery at the time they started the intervention; whilst it was difficult for them to know with any certainty, the majority suggested that starting the intervention earlier may have been beneficial, stating that IMT could have been introduced once they had recovered from the acute symptoms. Although this varied between individuals it was typically suggested that starting IMT around three months post-acute infection may be appropriate:

“*It is hard to say isn’t it… I think possibly a bit earlier but I think I still got the benefits from doing it now but I think*, *yeah maybe sort of a couple of months earlier but I think any earlier than that I was still having real issues with sort of fatigue so that might have impacted on sort of how well I would have been able to do it”* (P037).

Upon completion of the programme participants stated a desire to continue the training to help to maintain any perceived benefits, with some suggesting that they would purchase their own device if it was affordable:

“*I don’t know how much they [PrO2] cost*, *so I think if the cost was reasonable then yeah I would look at purchasing one… So I think yeah I think it would just depend on cost but*, *as I say*, *because I know that it really works…”* (P037)

For the most part, participants found that the programme was feasible and they were able to complete the training:

“*… it’s a reasonable amount of time*, *it’s not excessively long*, *yeah*, *I thought eight weeks was okay and*, *and three a week was okay*. *I wouldn’t have wanted it consecutive days because it*, *initially*, *where it was tiring me*, *it was*, *I wouldn’t have wanted to do it anymore”* (P064)

However, for some this was not feasible, with the primary barriers relating to time commitment required and finding the training somewhat tedious:

“*The three sessions sounds like a really easy target to hit but in amongst everything else*, *it was actually really hard to do it three times a week and what I was finding was sometimes I would leave it I would realise I’d left it four*, *five*, *six days…”* (P010)

“*On a negative side*, *I got a bit bored of it”* (P042)

Although compliance was generally good, some participants reported difficulty scheduling the sessions and remembering when to do the IMT, stating that a scheduling system or notifications would help to improve their compliance:

“*I should have set a reminder on my phone or a little chart or something but then sometimes I couldn’t remember when I last did it”* (P007)

## Discussion

The aim of this study was to explore individuals’ experiences of using IMT to support their recovery from COVID-19. The findings indicate that individuals experiencing persistent symptoms associated with COVID-19 felt unsupported in managing their condition and found it challenging to cope with the uncertainty and lack of understanding of the variety and severity of their symptoms. Although the symptoms described were poorly understood early in the pandemic, they are broadly comparable to the variety of symptoms we now understand to be associated with post-COVID-19 [4]. Previous research has also sought to understand the impact that these symptoms have on an individual’s lifestyle, including ADL, physical activity and work [9, 20]. As well as furthering this understanding, the current study suggests that an intervention, in this case IMT, may help to lessen this impact and help some individuals to return to their usual activities. Given the perceived lack of support and available rehabilitation strategies, participants valued the opportunity to discuss and have their condition recognised, receive support and be proactive about their recovery, regardless of the perceived benefit of the training. A similar qualitative study which explored the lived experiences of individuals with ‘long Covid’ also highlighted the symptom burden of COVID-19, the difficulties accessing support and a lack of understanding of COVID-19, which culminated in the suggestion that individuals with persistent COVID-19 symptoms would benefit from access to care from a multi-disciplinary team [21]. These findings are consistent with the suggestions made by participants in the current study when discussing the real world application of IMT in clinical practice.

IMT was associated with perceived improvements in respiratory symptoms, and exercise confidence and capacity which may also have resulted in increased levels of physical activity, including ADLs and work. Whilst participants reported that the intervention was feasible, there were some participants who found the training challenging, particularly those experiencing fatigue. This highlights an important consideration for future interventions using IMT in people recovering from COVID-19, given that persistent fatigue is commonly reported [22]. Future use of IMT in individuals recovering from COVID-19 should also consider the complexity of the condition and its varied symptomatology, recognising the requirement to tailor the training on an individual basis and provide support throughout.

The qualitative methods used in this study facilitated an in-depth exploration of individuals’ experiences of COVID-19 and the use of IMT in their recovery. Participation was voluntary, and recruitment was primarily conducted via online advertising. There is therefore potential for a self-selection bias towards individuals seeking specific rehabilitation strategies to support their recovery. The recruitment process may have resulted in a bias towards individuals who more actively engaged in social media. Recruitment also resulted in a predominantly female sample and there is growing evidence that being female is an independent risk factor for non-recovery from COVID, at least in hospitalised patients [23]; further research is therefore required to explore any potential sex differences. A confirmed positive test was not required to participate in the study due to the lack of testing available during the earlier stages of the pandemic. Nevertheless, the majority of participants reported having either a positive test or diagnosis based on clinical symptoms.

The generalisability of the results is not based on conventional statistical probability but rather on the detailed exploration of multiple perspectives from a diverse sample of individuals recovering from COVID-19 of varied age and recovery duration [24]. Finally, nine of the thirty-three pre-intervention interviews analysed have previously been analysed and reported on in a separate study exploring the impact of COVID-19 on physical activity. However, this analysis was conducted by a separate researcher and coded independently to specifically explore physical activity rather than to explore the impact of an IMT intervention [9].

In conclusion, this study highlights individuals’ experiences of the long-term impact of COVID-19 and the potential benefits of IMT. It is likely that IMT will be most effective as part of a wider rehabilitation strategy, taking a holistic approach to recovery, rather than as a standalone therapy. Further research is required to assess the long-term impact of IMT and the opportunities to tailor IMT based on individual symptoms and stage of recovery.

## Supporting information

S1 File COREQ checklist. (PDF)

S2 File Descriptive characteristics of participants. (XLSX)
